# A 2000–2023 dataset for measuring China’s biodiversity risk

**DOI:** 10.1016/j.dib.2026.112650

**Published:** 2026-05-06

**Authors:** Zhang-Hangjian Chen, Xiang Gao, Jin Rui Yang, Larisa Yarovaya

**Affiliations:** a School of Economics, Anhui University, Hefei 230601, China; b Research Center of Finance, Shanghai Business School, Shanghai 200235, China; c Southampton Business School, University of Southampton, Southampton, SO17 1BJ, UK

**Keywords:** Chinese market, Biodiversity risk, Machine learning, BERT model

## Abstract

The increasing frequency of extinctions of biological populations has important implications for related sectors. Consequently, the risks associated with biodiversity are receiving increasing attention and are being recognized as entirely new risk factors. To understand the drivers of biodiversity risk, it is crucial to measure biodiversity risk at multiple levels, especially in developing countries. Using machine learning and text mining methods, we measure the biodiversity risk of the Chinese market from 2000 to 2023 from the perspectives of macro-government, meso‑industry, and micro-firms, by analysing official news media texts, related fund-holding data, and listed firms’ annual reports. We construct a macro-level index derived from the sentiment and frequency of biodiversity-related discourse in official media, including the China Environment News and the CCTV News, from 2013 to 2023. We also construct a meso-level industry risk exposure indicator, calculated as the deviation of biodiversity-themed public funds’ holdings from market portfolio weights across 58 sectors. In addition, we develop micro-level firm-specific metrics, based on the frequency and sentiment analysis of biodiversity sentences in the annual reports of 5606 A-share listed firms, 2000–2023, using an improved BERT model for Chinese text. These indicators provide researchers, policymakers, and financial practitioners with a foundational resource for empirically investigating the economic and financial implications of biodiversity risk in China.

Specifications Table

**Table:** 

Subject	Economics, Econometrics and Finance.
Specific subject area	Climate and Environmental Finance.
Type of data	Table (.xlsx file)
Data collection	The biodiversity risk indicators are constructed using data from various sources, including annual reports of listed firms, news articles from China Environment News and CCTV News. Annual reports and news texts are obtained from official websites using web crawlers. Basic information about listed firms, such as office location and industry classification, is gathered from the CSMAR and WIND databases. Biodiversity-themed fund data are sourced from DailyFund.com, with fund holding data also sourced from the CSMAR and WIND databases. To analyze biodiversity risks, a comprehensive dataset is compiled by extracting biodiversity-related sentences from the collected texts. The analysis focuses on three dimensions of biodiversity risk: macro (government-level assessments), *meso* (industry-level evaluations), and micro (firm-level insights). A news-based biodiversity sentiment index is defined as the number of positive biodiversity-related sentences minus the number of negative biodiversity-related sentences. At the meso‑level, a holding-based score for biodiversity-related public funds is constructed, measured as the difference between an industry’s market capitalization weight and its weight in fund portfolios. Finally, a firm-level biodiversity score is defined following a similar sentiment-based rule. These values represent the different tiers of biodiversity risk.
Data source location	Country: China.
Data accessibility	Repository name: Mendeley Data Data identification number: 10.17632/ny5x3bkd56.1 Direct URL to data: https://doi.org/10.17632/ny5x3bkd56.1 Instructions for accessing these data: Saved but unpublished with the following citation: Chen, Zhang-Hangjian; Gao, Xiang; Yang, JinRui; Yarovaya, Larisa (2024), “A dataset to measure China biodiversity risk”, Mendeley Data, V1, doi:10.17632/ny5x3bkd56.1.
Related research article	Chen, Zhang-Hangjian., Derwall, Jeroen, Gao, Xiang, Koedijk, Kees (2024), “Biodiversity Risk in Emerging Economies: Evidence from China”, Working Paper.

## 1 Value of the Data

• These data provide a comprehensive measure of biodiversity risk across three dimensions: macro (government), *meso* (industry), and micro (firms). This offers a multi-perspective view of biodiversity risk in China. Unlike previous approaches, our data facilitates clustering in categorical domains such as time, city, and industry. This allows the index to be aligned with a wide range of relevant studies.
• The macro data is derived from news articles in Chinese mainstream media between 2013 and 2023, covering 5394 trading days. Meanwhile, the meso‑data comes from over 40 funds related to conceptual themes such as “bioprotection” listed between 2015 and 2023. The firm-level data comprise biodiversity risk indicators extracted from the annual reports of 5606 firms listed on the Shanghai, Shenzhen, and Beijing stock exchanges between 2000 and 2023. Although some researchers have used similar sources of information [[1], [2], [3], [4], [5]], there is currently no publicly available dataset sufficient to capture biodiversity risk across different domains in the Chinese market. This dataset can fill this gap. Researchers, business managers, financial analysts, and policymakers can use this dataset to analyze the macro- and micro-level impacts of biodiversity risks on the economy and society. This facilitates proactive strategies to mitigate the negative effects of biodiversity risks across macroeconomic systems, financial markets, enterprises, and institutions, among others.
• These data can be matched with most relevant studies, enabling researchers to analyze biodiversity risk from multiple perspectives, including macroeconomic, financial market, and corporate impacts. The dataset’s ability to cluster information over time by city and industry enables targeted studies to be conducted, such as the assessment of regional biodiversity risks or sector-specific vulnerabilities. This dataset can also be used by researchers to explore relationships between biodiversity risks and economic indicators, formulate risk-mitigation strategies, and investigate trends in biodiversity risks over time. Its public accessibility facilitates interdisciplinary research and comparative analysis, leading to new insights into biodiversity risk management.

## 2 Background

In recent years, climate change has been at the center of global attention, with significant progress being made in the study of climate risk. Scientists have explored issues such as greenhouse gas emissions, rising temperatures and sea levels, and have predicted the impacts of these changes on human societies. However, the extant empirical research on biodiversity risk is still in its early stages of expansion [[8], [9], [10], [11], [12], [13]]. This unbalanced research may have negative consequences, as biodiversity is a crucial foundation for ecosystem stability and human well-being [14].

Biodiversity encompasses the diversity of ecosystems, species and genes that together maintain ecological balance and environmental health. Human societies depend on biodiversity in many ways [5]. For example, agriculture relies on a diversity of crop varieties and pollinators, drug development utilizes a wide range of plant and animal chemicals, and fisheries depend on healthy marine ecosystems. Additionally, biodiversity is crucial for ecological services such as climate regulation, water purification, maintaining soil fertility and providing flood and wind protection. However, a number of major loss events have occurred around the world as a result of neglecting biodiversity risks [15].

A case in point is the destruction of agriculture by locust plagues. Swarms of locusts have emerged in several regions around the globe, particularly in Africa and Asia, posing a serious threat to food security. The locust plague that hit East Africa in 2020, for example, destroyed large areas of farmland and disrupted the food supply for millions of people. The frequency and severity of locust plagues are partly attributable to climate change and habitat destruction, both of which are also important causes of biodiversity loss. The event highlighted the direct economic and social consequences of failing to maintain ecosystem balance and biodiversity.

Another case is the disappearance of coral reefs. Not only are coral reefs important habitats for marine life, but they also play a key role in protecting coastal communities from wind and waves. Coral reefs are rapidly disappearing globally due to ocean acidification, overfishing and pollution. For example, Australia’s Great Barrier Reef is currently experiencing severe bleaching. This has led to the death of large numbers of marine organisms and damaged marine ecosystems, directly affecting local economies that depend on tourism and fisheries.

Therefore, the scientific community and policymakers must be aware of the importance of biodiversity risks and increase their research and conservation efforts in this area. Global environmental challenges can be addressed more effectively by integrating consideration of climate change and biodiversity risks. This helps to ensure the sustainable development of the planet and the long-term well-being of humankind.

## 3 Data Description

Following Engle et al [6], we create a biodiversity risk index by extracting news stories about biodiversity risk from newspapers or news reports on biodiversity-related topics. These indicators measure the macro-level biodiversity risk, including the attention and sentiment biases of the official media and the general public towards biodiversity loss concerns. Regarding data on news-based biodiversity risk, the aggregate attention of the government, mainstream professional media, and the public to biodiversity is calculated from a macro perspective, using data from CCTV News, China Environment News, and the Baidu Search Index, respectively. Daily content text data is sourced from the CCTV News, daily content text from the China Environment News, and daily searches for biodiversity keywords in the Baidu search engine (https://index.baidu.com/v2/index.html). CCTV News is the most-watched and influential television news program in China (https://www.cctv.com). It serves as an important barometer of Chinese politics, representing the Chinese government’s position and attitude on relevant issues. The China Environment News (https://epaper.cenews.com.cn/) is an official national environmental protection newspaper under the supervision of the Ministry of Ecology and Environment of the People’s Republic of China, and always adheres to the goal of preventing and controlling pollution, improving the ecology, promoting development, and benefiting the people. Baidu is the world’s largest Chinese search engine, and the Baidu Search Index is a data-sharing platform based on Baidu’s massive Internet user behavior data.

This study measures meso‑level biodiversity risk by constructing an indicator based on the deviation between an industry’s market capitalization-weighted share and its share in the portfolios of biodiversity-themed funds.

The micro-level biodiversity risk analysis is conducted using the annual reports of 5606 Chinese listed firms from 2000 to 2023. The number of biodiversity-related sentences in each report is counted as an attention indicator. Subsequently, an improved BERT model is employed to perform sentiment analysis on these sentences, categorizing them into positive, negative, or neutral groups. Based on this sentiment categorization, the biodiversity risk exposure level for each firm is calculated. The contents of the repository are presented in Table 1.

**Table 1:** Overview of the content of the repository.

File name	Sheet name	Sampling period	Frequency	Variables	Data source
Annual report-based biodiversity risk exposure.xlsx	biodiversity risk	2000–2023	Yearly	Stock code	*The text of the annual report is taken from the Cninfo website* (http://www.cninfo.com.cn/new/commonUrl/pageOfSearch?url=disclosure/list/search&lastPage=index).
				Date	
				Company id	
				Security id	
				Industry code	
				Office zip	
				Province code	
				City code	
				$Frq_{Bio,it}^{AnuRep}$	
				$Sti_{Bio,it}^{AnuRep}$	
Funds holding-based biodiversity risk exposure.xlsx	biodiversity risk	2011–2023	Yearly	Industry	*Fund information is sourced from DailyFund.com* (https://fund.eastmoney.com/001856.html?spm=search). Fund holdings data sourced from CSMAR database.
				Industry code	
				Year	
				$W_{m}^{I}$	
				$W_{f}^{I}$	
				$BioRisk^{I}$	
News-based biodiversity risk exposure.xlsx	Baidu	2006.06–2024.06	Monthly	Date	https://index.baidu.com/
				$Frq_{Bio}^{Baidu}$	
	China Environmental News	2013.09.02–2023.12.29	Daily	Date	https://epaper.cenews.com.cn/html/2025/20251212/20251212_001/20251212_001_5609.html#0
				$Frq_{Bio}^{Cenews}$	
				$Sti_{Bio}^{Cenews}$	
	CCTV News	2006.09.01- 2024.06.07	Daily	Date	https://tv.cctv.com/lm/xwlb/
				$Frq_{Bio}^{CCTV}$	
				$Sti_{Bio}^{CCTV}$	

## 4 Experimental Design, Materials and Methods

For data collection, the annual reports of listed firms, the original text of China Environment News, and the text of CCTV News are obtained from official websites using web crawlers. Basic information on the listed firms, including their office locations and industries, is obtained from the CSMAR and WIND databases.

Data processing follows the five steps as described below:

Step 1: A total of 38,260 articles on biodiversity is retrieved from the China Knowledge Network (CNN). The keywords from these articles are used as biodiversity keywords. Following manual screening, 451 biodiversity-related words are selected as keywords for biodiversity statements.

Step 2: Text segmentation is performed using regular expressions, with punctuation as the boundary, to identify the text content and obtain the body part that meets the requirements.

Step 3: The text processing procedure involves several steps. First, all sentences are scanned for biodiversity keywords and filtered to remove irrelevant content. Second, a representative sample of sentences is drawn from the annual report and news datasets. Third, each sentence is assigned a sentiment score, e.g., positive=1, neutral=0, negative=−1. To enhance reliability, a manual annotation stage is conducted by nine industry experts. The adopted cross-labeling approach ensures that each sentence in the training and validation sets is reviewed by three independent annotators.

Step 4: A pre-trained BERT model (hfl/chinese-roberta-wwm-ext), developed by HFL based on the RoBERTa architecture [7], is employed. First, the model is used to generate a validation report. After picking out the best results, sentiment evaluation analysis is performed separately on annual reports and news articles. Finally, sentiment labels are assigned to all sentences based on this analysis.

**Step 5:** Next, we construct the index according to the following three steps.

(1) Calculating the macro biodiversity risk indicator. The number of biodiversity-related sentences in CCTV News and China Environment News from 2013 to 2023 is recorded. $Frq_{Bio,t}^{CCTV}$ represents the number of sentences related to biodiversity in CCTV News at time t, and the similar indicator is $Frq_{Bio,t}^{Cenews}$, denoting the number of sentences related to biodiversity in China Environment News on time t. If there is no relevant sentence, we count it as 0.

And then, the biodiversity sentiment index is calculated based on the sentiment tendency of biodiversity-related sentences in the above News from 2013 to 2023. $Label_{Bio,it}^{CCTV}$ denotes the sentiment label number of the i th sentence related to biodiversity based on CCTV news, and similarly $Label_{Bio,it}^{Cenews}$ denotes the sentiment label number of the i th sentence related to biodiversity based on CCTV news. $Sti_{Bio,it}^{CCTV}$ denotes the sentiment index of biodiversity based on CCTV news at time t. When there is no sentence related to biodiversity, we count it as a null value.

(1) $$Sti_{Bio,t}^{CCTV}=\sum_{i=1}^{n}Label_{Bio,i}^{CCTV},$$

(2) $$Sti_{Bio,t}^{Cenews}=\sum_{i=1}^{n}Label_{Bio,i}^{Cenews}.$$

(2) The meso‑level biodiversity risk indicator is calculated by aggregating the holding scores of biodiversity-related public funds at the sector level. An annual industry-level biodiversity risk exposure indicator is then constructed using information on the industry exposures of 40 environmental and biodiversity funds:

(3) $$BioRisk_{t,f}^{I}=w_{t,m}^{I}-w_{t,f}^{I}$$

where $w_{t,m}^{I}$ is the weight of industry I in the market portfolio at time t, $w_{t,f}^{I}$ is the weight of industry *I* in fund *f* at time *t*. The biodiversity risk exposure indicator for industry *I* is $\mathrm{BioR}\mathrm{is}k_{t}^{I}=\sum_{f}^{F}\mathrm{BioR}\mathrm{is}k_{t,f}^{I}/N$*,* with *N* being the number of funds holding industry *I* stocks. A larger $\mathrm{BioR}\mathrm{is}k_{t}^{I}$ indicates that funds underweight industry *I* relative to its weight in the market portfolio, i.e., industry *I* is more exposed to biodiversity risk. The industry classification is based on the 2012 edition of the CSRC (China Securities Regulatory Commission) Industry Classification, and we calculate biodiversity risk exposure indicators for 58 secondary industries.

*(3)* Finally, this study turns to the measurement of biodiversity risk at the firm level. We construct several alternative measures of biodiversity risk based on the text of the annual reports of 5606 listed firms for the period 2000 to 2023. First, the number of sentences containing at least one biodiversity term in the text of each listed firm’s annual report ($Frq_{Bio,it}^{AnuRep}$) is counted as an indicator of biodiversity concern at the micro-firm level. A firm-level biodiversity risk exposure metric is then developed, incorporating information from positive ($Frq_{Bio,it}^{Pos}$), neutral ($Frq_{Bio,it}^{Neu}$), and negative ($Frq_{Bio,it}^{Neg}$) sentences. An improved BERT model is applied to sentence classification. Chinese text is complex and diverse, and traditional sentiment analysis tools, such as simple, plain Bayesian classifiers, can be misled by its ambiguities. The BERT model can accurately capture the meaning of words across contexts by simultaneously considering the pre- and post-contextual information to each word through a bidirectional encoder. In addition, the BERT model significantly improves accuracy and specialization by training on a large-scale Chinese corpus and then fine-tuning it for a specific task. Summing up, we classify the sentiment of 369,762 sentences containing biodiversity terms as 92.2 % neutral, 7.1 % positive, and 0.7 % negative. $Frq_{Bio,it}^{AnuRep}$ denotes the number of sentences related to biodiversity in the annual report of the listed firm i in year t. $Label_{Bio,it}^{AnuRep}$ denotes the number of sentiment labels based on the i-th sentence related to biodiversity in the annual report of listed firm *i*. Finally, a company’s biodiversity risk exposure ($Sti_{Bio,it}^{Action}$) is measured by the difference between the number of negative sentences related to biodiversity and the number of positive sentences related to biodiversity:

(4) $$Sti_{Bio,it}^{AnuRep}=\sum_{i=1}^{n}Label_{Bio,it}^{AnuRep}$$

## Limitations

This dataset measures biodiversity risk in the Chinese market, which may be complicated by China’s distinct geographical and cultural characteristics in the current era [16]. Additionally, we employ a machine learning approach to analyse the sentiment expressed in Chinese text. While the out-of-sample classification accuracy exceeds 80 %, the algorithm may lack explainability and contain unavoidable biases due to sample selection and data curation.

## Ethics Statement

The authors have read and followed the ethical requirements for publication in Data in Brief and confirmed that the current work does not involve human subjects, animal experiments, or any data collected from social media platforms.

## CRediT Author Statement

All authors contributed equally to idea conceptualization, investigations, and writing the manuscript.

## Data Availability

Mendeley DataA dataset to measure China biodiversity risk, Mendeley Data, V1 (Original data)
